# Rare complication of rheumatoid arthritis: Charcot Neuro-osteoarthropathy

**DOI:** 10.1186/s12891-024-07424-y

**Published:** 2024-04-29

**Authors:** Zhiyuan Luo, Xinxiang Ding, Yu Yuan, Lei Hou

**Affiliations:** 1 The Eighth Clinical Medical College of Guangzhou University of Chinese Medicine, Foshan, Guangdong China; 2 Foshan Hospital of Traditional Chinese Medicine, Foshan, Guangdong China

**Keywords:** Charcot Neuro-osteoarthropathy, Rheumatoid arthritis, Molecular mechanism;diagnosis, Treatment, Case report

## Abstract

**Background:**

Rheumatoid arthritis (RA) is an autoimmune disease.However, there are few cases of Charcot Neuro-osteoarthropathy (CN) caused by rheumatoid diseases in clinical reports. It is not easy to pay attention to the diagnosis of CN in the complications of rheumatoid disease, which greatly increases the probability of misdiagnosis and missed diagnosis. This case reported a rare complication of rheumatoid arthritis, Charcot arthritis, and the molecular mechanism and diagnosis and treatment of CN caused by RA were systematically discussed.

**Case presentation:**

The patient, a 79-year-old woman, was hospitalized due to bilateral shoulder pain, limited activity for half a year, aggravated for 4 months to the hospital. During this period, the symptoms did not improve after treatment with acupuncture and Chinese medicine. The patient was previously diagnosed with rheumatoid arthritis for more than 3 years and intermittent irregular use of methylprednisolone and methotrexate for 2 years. She had a history of osteoporosis. Physical examination: symmetrical malformed swelling of the finger joints of both hands; Bilateral supraspinatus and deltoid muscle atrophy, tenderness at the acromion, and attachment of the long head tendon of the biceps brachii were observed. The left Dugas test and the right Dugas test were positive.Blood test: anti-cyclic citrullinated peptide antibody (A-CCP) 33.10U/ml (normal range: 0-5RU/ml); antinuclear antibody quantification (ANA) 47.40AU/ml (normal range: Negative or < 32); anti-double stranded DNA IgG antibody quantification (dsDNA) 31.00 IU/ml (normal range: 0-100 IU/ml); D-Dimer 6.43 µg/ml (normal range: 0–0.5 mg/L); erythrocyte sedimentation rate (ESR) was 27 mm/h (normal range: < 20 mm/60 min). C-reactive protein (CRP) 39.06 mg/L(0.068-8 mg/L).MRI 3.0 T enhancement of bilateral shoulder joints, cervical spine and thoracic spine showed: 1.Large bone destruction, cartilage injury, multiple effusion, synovitis, obvious on the right side. 2.Intervertebral disc degeneration, cervical 3/4, 4/5, 5/6, 6/7 disc herniation, with cervical 3/4 obvious, posterior central herniation;

**Conclusions:**

Rheumatoid arthritis complicated with Charcot's joint is rare. Clinically, patients with rheumatoid diseases should not ignore Charcot's joint complications because of rareness. Early blood inflammatory markers, neuro electrophysiology, and imaging MRI of rheumatoid CN are of great significance for the diagnosis of this mild or early neurovascular inflammation. Early diagnosis and treatment are helpful to prevent further joint injury. The clinical diagnosis, treatment, and molecular mechanism of osteolysis in RA and peripheral sensory nerve injury remain to be further revealed.

## Background

Rheumatoid arthritis (RA) is an autoimmune disease characterized by symmetrical joint pain with swelling, stiffness, and deformity, which is common in the hands, feet, and knees. Common complications include infection, respiratory disease, osteoporosis, and cardiovascular disease [1]. However, there are few cases of Charcot Neuro-osteoarthropathy (CN) caused by rheumatoid diseases in clinical reports. It is not easy to pay attention to the diagnosis of CN in the complications of rheumatoid disease, which greatly increases the probability of misdiagnosis and missed diagnosis. This case reported a rare complication of rheumatoid arthritis, Charcot arthritis, and the molecular mechanism and diagnosis and treatment of CN caused by RA were systematically discussed.

## Case report

**Fig. 1:**
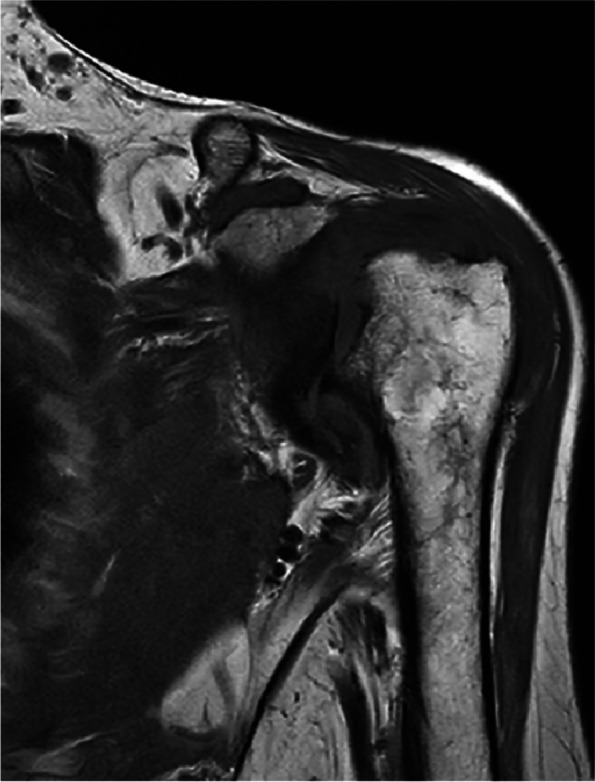
Coronal MRI of the left shoulder joint

**Fig. 2:**
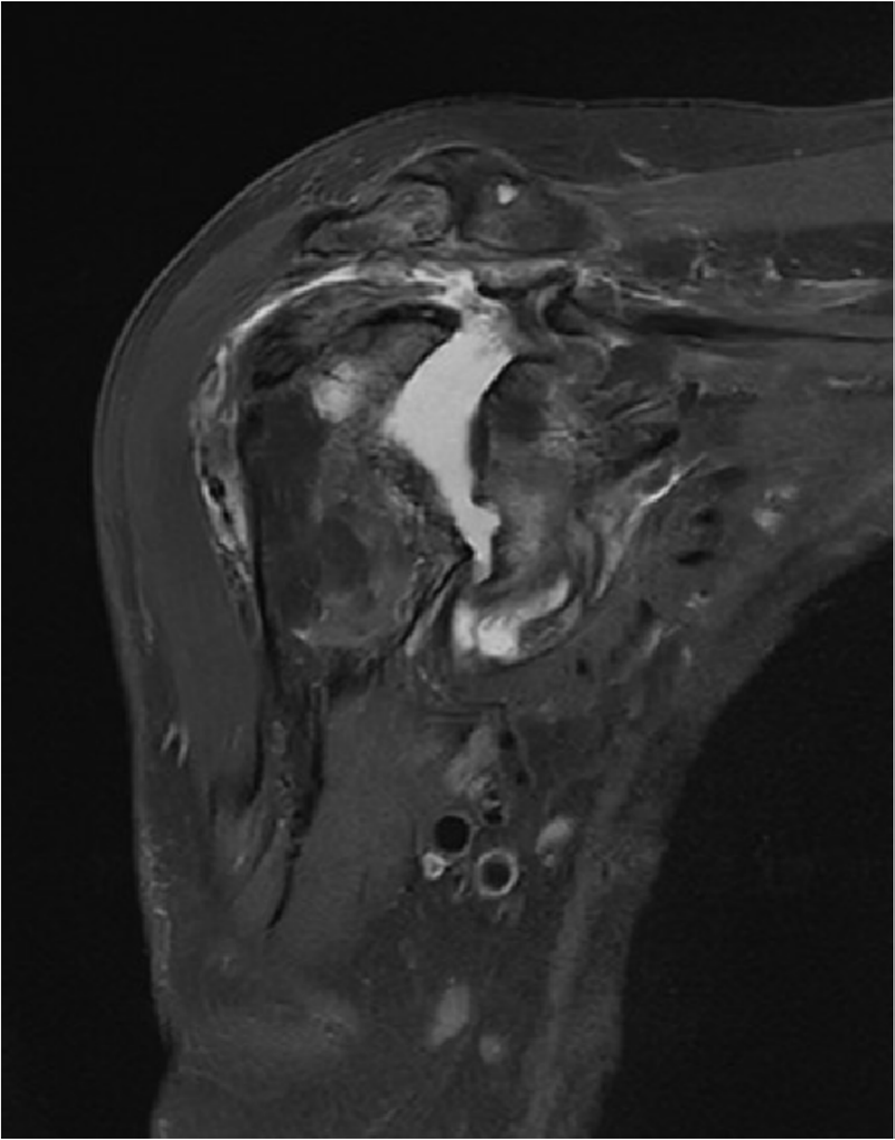
Coronal MRI of the right shoulder joint (fat-suppression sequence)

**Fig. 3:**
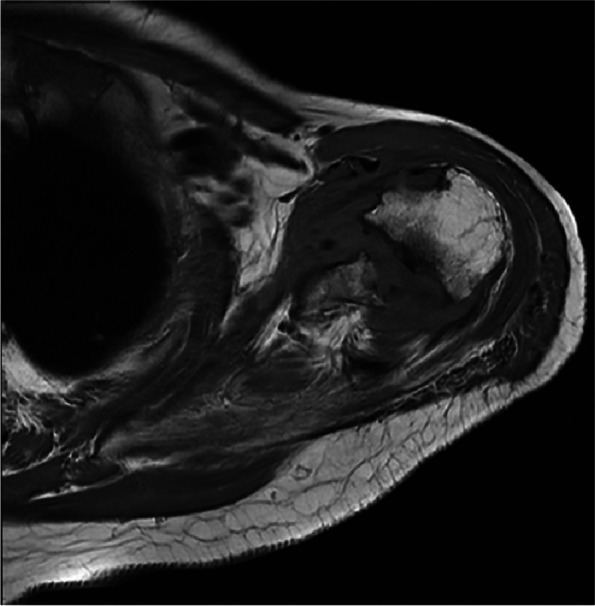
Transverse MRI of the left shoulder joint

**Fig. 4:**
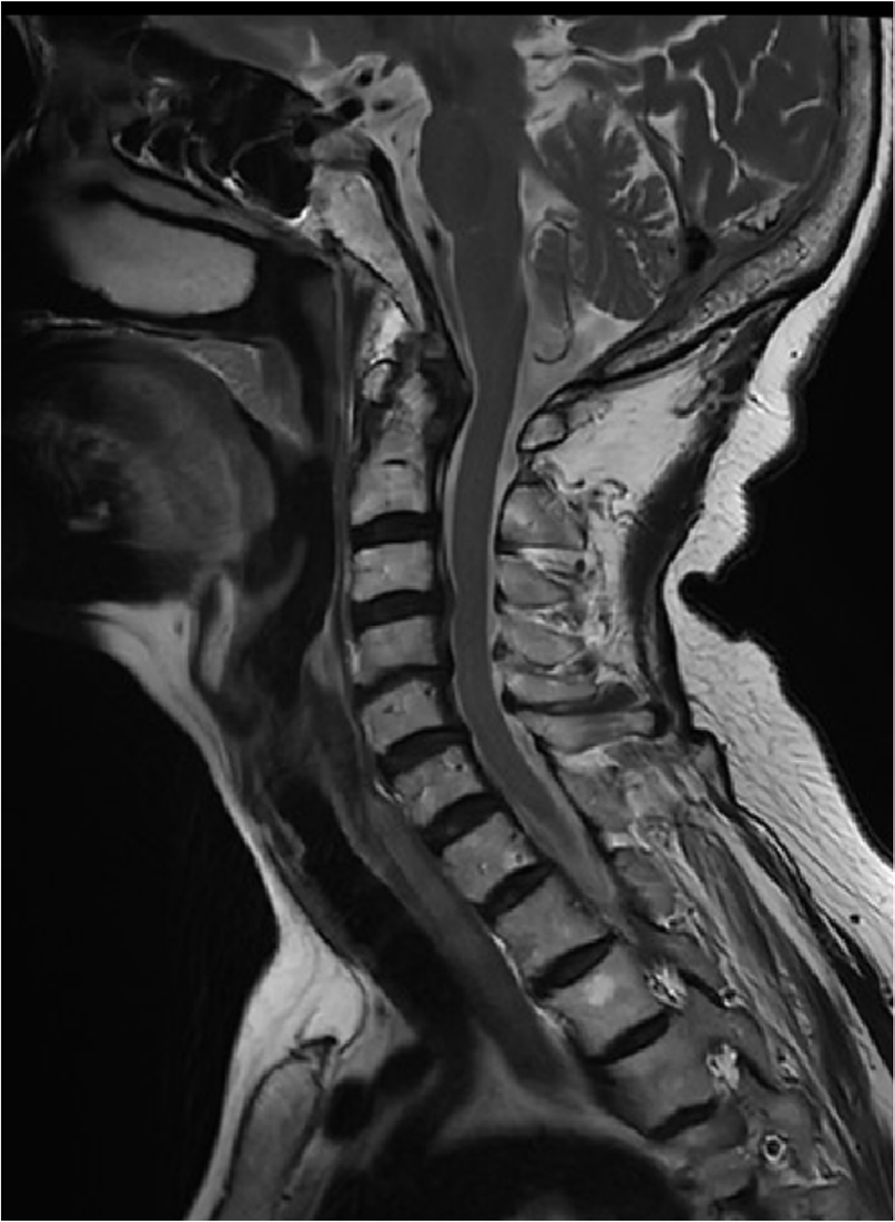
Sagittal MRI of cervical spine

The patient, a 79-year-old woman, was hospitalized number 522702 due to bilateral shoulder pain, limited activity for half a year, aggravated for 4 months to the hospital. The patient began to have bilateral shoulder joint pain and limited activity without obvious inducement half a year ago, without limb weakness, numbness, or neck pain. During this period, the symptoms did not improve after treatment with acupuncture and Chinese medicine in the outpatient department of the hospital. The patient was previously diagnosed with rheumatoid arthritis for more than 3 years and intermittent irregular use of methylprednisolone (2 mg/d,taken irregularly)and methotrexate(12.5 mg/d, taken irregularly) for 2 years. She had a history of osteoporosis, denied diabetes and syphilis, and had no history of trauma or surgery. Physical examination: symmetrical malformed swelling of the finger joints of both hands; there was no swelling or ecchymosis in the bilateral shoulders. Bilateral supraspinatus and deltoid muscle atrophy, tenderness at the acromion, and attachment of the long head tendon of the biceps brachii were observed. The active range of motion of both shoulders: flexion 30 degrees(Reduce by 60 degrees), extension 15 degrees(Reduce by 30 degrees), abduction 30 degrees(Reduce by 60 degrees); the passive range of motion of the left shoulder joint: flexion 90 degrees, abduction 90 degrees, extension 15 degrees(Reduce by 30 degrees); the passive range of motion of the right shoulder joint: 60 degrees of flexion(Reduce by 30 degrees), 60 degrees of abduction(Reduce by 30 degrees), and 15 degrees of extension(Reduce by 30 degrees). The left Dugas test and the right Dugas test were positive, the muscle strength of both hands was graded as 4, indicating weakened muscle tension. Additionally, VAS (Visual Analogue Scale) is 3 score in shoulders. Value of DASH (Disabilites of the Shoulder and Hand) is 54.2.Blood test: anti-cyclic citrullinated peptide antibody (A-CCP) 33.10U/ml (normal range: 0-5RU/ml); antinuclear antibody quantification (ANA) 47.40AU/ml(normal range: Negative or < 32); anti-double stranded DNA IgG antibody quantification (dsDNA) 31.00 IU/ml (normal range: 0-100 IU/ml); treponema pallidum antibody was negative; D-Dimer 6.43 µg/ml (normal range: 0-500ug/L); erythrocyte sedimentation rate (ESR) was 27 mm/h (normal range: < 20 mm/h). C-reactive protein (CRP) 39.06 mg/L(0.068-8 mg/L). There are no abnormalities found in thyroid function, blood calcium and phosphorus levels, routine urine tests, and the parathyroid ultrasound did not reveal any abnormalities in the parathyroid glands. Anteroposterior X-ray of shoulder joint: hyperplasia and hardening of bone at the edge of shoulder joint, narrowing of shoulder joint space. Figures 1, 2 and 3 MRI 3.0 T enhancement of bilateral shoulder joints showed: the correspondence between the shoulder joints on both sides was unstable, the joint space was different in thickness and narrowness, the bone collapse and defect of the medial humerus head on both sides, the glenoid lip was unclear, and the surrounding bone edema presented long T1 and T2 abnormal signal shadows. There was thickening and swelling of the bursae of the shoulder joint capsule on both sides. There was a lot of fluid accumulation in the joint cavity, and the surrounding synovium thickened obviously, especially on the right side. The enhanced scan showed patchy enhancement of the synovium of the shoulder joint on both sides, and patchy enhancement of the adjacent bone structure with blurred edges. Bilateral acromioclavicular joint hyperplasia. Diagnosis:1. Large bone destruction, cartilage injury, multiple effusion, synovitis, obvious on the right side, combined with medical history, in line with the changes in the Charcot joint. 2. Degenerative changes in the bilateral acromioclavicular joints. Figure 4 Cervical spine and thoracic spine MRI 3.0 T showed the following: 1. Cervical degenerative changes, cervical 4 instability; 2. Intervertebral disc degeneration, cervical 3/4, 4/5, 5/6, 6/7 disc herniation, with cervical 3/4 obvious, posterior central herniation; 3. Slight stenosis of the cervical 4/5 plane spinal canal. Diagnosis: 1. CN 2. RA.Treatment: Due to the patient's refusal of surgery and request for conservative management, they were prescribed methylprednisolone tablets (4 mg, qd), Tripterygium wilfordii glycosides tablets (10 mg, tid), calcitriol soft capsules (0.25 µg, qd), and electroacupuncture therapy.

## Discussion

CN is a joint disease secondary to neurosensory loss. French doctor Jean-Martin Charco first discovered and described it in 1868 [2]. The primary etiologies of cervical neuropathy (CN) encompass conditions such as diabetes mellitus, syringomyelia, spinal dural arteriovenous fistula, acute trauma, syphilitic infections, paraplegia, and spinal cord tumors. Additionally, the administration of corticosteroids, whether orally or through intra-articular injection, may precipitate the development of this condition.The main feature is that the degree of pain is not consistent with the pathological nature of the disease. It can be divided into acute early stage, acute late stage, and chronic stable stage according to disease progression. Charcot's shoulder arthritis has been mainly described as a neurological disease secondary to cervical syringomyelia. Although there is still a lack of basic experiments and sufficient clinical evidence to show that CN is directly related to rheumatoid immune diseases, a few scholars have reported the relationship between the two. In 1987, Carl W. Hardin, M.D. reported the possibility of rheumatoid arthritis caused by Charcot's joint disease[3]. In 2014, Benjamin J. Grear, MD reported that Charcot foot could not find any other possibility except rheumatoid factor and suggested that there should be a certain correlation between the two[4]. In this case, rheumatoid disease may also be an independent risk factor for Charcot joints because the probability of actual observation caused by misdiagnosis and missed diagnosis is far underestimated.

CN is not a primary disease. Its more common primary diseases include syringomyelia, diabetes, acute trauma, syphilis, and intra-articular injection of hormones, which can cause the disease. Diabetes is the most common cause [5]^.^ There is evidence that RA can cause uncommon peripheral sensory nerve axon damage, and nerve biopsy can show different histopathological features [6]. The pathological mechanism of peripheral nerve injury caused by RA may be related to anti-endothelial cell antibody (AECA), chemokine CX3CL1 (FKN), interleukin family 17 (IL-17), and other inflammatory factors, which cause inflammatory nerve injury of arterial blood vessels in joint sites [7–9]. The most classic mechanisms of CN are the neurotrauma theory and neurovascular theory. The neurotrauma theory holds that severe peripheral neuropathy leads to a lack of protective sensation and excessive mechanical activity leads to bone stress injury. The neurovascular theory holds that autonomic neuropathy appears in arteriovenous communication branches, and local blood flow increases, resulting in osteolysis. The common pathology of the two theories is joint inflammation. Inflammatory factors such as Tumornecrosisfactor-α( tumor necrosis factor-α (TNF-α), IL-1, and IL-6 act on the OPG/RANKL/RANK signal transduction pathway, inducing an imbalance in osteocyte autophagy and mitophagy to destroy the balance of bone turnover, which may be the key to the molecular mechanism of RA complicated with CN [10–14]. In this case, the image of CN patients with osteoporosis showed osteolysis and edema signals in the surrounding tissue, and the inflammatory indexes were significantly increased. It was not only related to CN but also did not exclude that RA was related to the erosion of surrounding connective tissue, which led to limited activity, which seemed to be different from the CN reported in the literature. RA can induce an imbalance between autophagy and mitophagy, leading to the secretion of proinflammatory factors and RANKL in fibroblast-like synovial cells, which mediates the activation of bone resorption, destroys the balance of bone turnover, promotes more active osteoclasts and aggravates osteoporosis, osteolysis, and connective tissue damage [15–17].

The diagnostic standard of RA complicated with CN is still unclear. Acute early: Due to the acute release of trauma and proinflammatory cytokines, the expression of RANKL is increased, which mediates clinical inflammation and leads to osteoclast maturation and osteolysis. Joint swelling, effusion, and fatigue in acute exacerbation [12]. RA invading sensory nerves and connective tissue can restrict joint movement through adhesion, causing either mild or painless limitations. X-ray results typically appear normal or slightly altered. However, the sensitivity of X-rays at this stage is not high, which often leads to missed diagnoses and misdiagnosis. Early MRI examination is a sensitive method for CN showing subchondral bone marrow edema. EMG and MCV tests may show outliers. Biopsy can also accurately diagnose early changes [18, 19]. Acute period: Joint swelling subsided, and the joint fluid cell composition was mainly yellow, sticky lymphocytes that easily coagulated. Mild bone damage, many loose bodies, and bone hyperplasia in the joints can be accompanied by subluxation and dislocation of the joints, palpation, and broken bone sensation. X-ray at this stage can show a wide range of injuries and changes. Chronic stable period: joint effusion bone deformity with dislocation and subluxation, abnormal movement, bone friction sound, and bone friction sense. X-ray showed fracture healing, hardening, and remodeling [20]. It also needs to be distinguished from the following diseases: other types of CN, degenerative joint disease, traumatic arthritis, joint tuberculosis, rheumatoid arthritis, suppurative arthritis, etc. It can be identified by primary underlying diseases, as well as specific laboratory tests, pathological examinations, and clinical symptoms and signs.

The primary disease should be diagnosed and treated first. Due to the lack of neurotrophic innervation of the affected joints, early joint plaster or bracket fixation and braking can greatly prevent the occurrence of deformity [21]. High-dose aspirin, nonsteroidal anti-inflammatory drugs, and methylprednisolone, as well as specific TNF-α antagonists and other anti-RA and anti-inflammatory drugs, can activate the RANKL/OPG pathway to protect bone tissue and effectively alleviate inflammation. In addition, some scholars believe that biphosphonates can block the development of acute Charcot arthropathy [22] and that electromagnetic stimulation can reduce the occurrence of Charcot joint deformity [23]. Late surgical treatment: Joint compression fusion and amputation can be considered when long-term conservative treatment is ineffective, but the success rate of bone fusion is very low. Joint replacement In recent years, Charcot joint replacement has gradually increased, and the shoulder joint has brought hope for Charcot joint disease [24]. However, there is still a lack of clinical evidence on the effect of CN shoulder replacement. Due to the complications of joint replacement, such as periprosthetic fracture, prosthesis loosening, and infection, there are many secondary operations, and the operation should be cautious [25].

## Summary

Rheumatoid arthritis complicated with Charcot's joint is rare. Clinically, patients with rheumatoid diseases should not ignore Charcot's joint complications because of rareness. Early blood inflammatory markers, neuro electrophysiology, and imaging MRI of rheumatoid CN are of great significance for the diagnosis of this mild or early neurovascular inflammation. Early diagnosis and treatment are helpful to prevent further joint injury. The clinical diagnosis, treatment, and molecular mechanism of osteolysis in RA and peripheral sensory nerve injury remain to be further revealed.

## Data Availability

If someone wants to request the data from this study,he/she should contact Zhiyuan Luo(189939454@qq.com).
